# The 3D-architecture of individual free silver nanoparticles captured by X-ray scattering

**DOI:** 10.1038/ncomms7187

**Published:** 2015-02-04

**Authors:** Ingo Barke, Hannes Hartmann, Daniela Rupp, Leonie Flückiger, Mario Sauppe, Marcus Adolph, Sebastian Schorb, Christoph Bostedt, Rolf Treusch, Christian Peltz, Stephan Bartling, Thomas Fennel, Karl-Heinz Meiwes-Broer, Thomas Möller

**Affiliations:** 1 Institute of Physics, University of Rostock, Universitätsplatz 3, 18055 Rostock, Germany; 2 IOAP, Technische Universität Berlin, Hardenbergstraße 36, 10623 Berlin, Germany; 3 Linac Coherent Light Source, SLAC National Accelerator Laboratory, 2575 Sand Hill Road, Menlo Park, California 94025, USA; 4 PULSE Institute, Stanford University and SLAC National Accelerator Laboratory, 2575 Sand Hill Road, Menlo Park, California 94025, USA; 5 FLASH, DESY, Notkestraße 85, 22607 Hamburg, Germany

## Abstract

The diversity of nanoparticle shapes generated by condensation from gaseous matter reflects the fundamental competition between thermodynamic equilibration and the persistence of metastable configurations during growth. In the kinetically limited regime, intermediate geometries that are favoured only in early formation stages can be imprinted in the finally observed ensemble of differently structured specimens. Here we demonstrate that single-shot wide-angle scattering of femtosecond soft X-ray free-electron laser pulses allows three-dimensional characterization of the resulting metastable nanoparticle structures. For individual free silver particles, which can be considered frozen in space for the duration of photon exposure, both shape and orientation are uncovered from measured scattering images. We identify regular shapes, including species with fivefold symmetry and surprisingly large aspect ratio up to particle radii of the order of 100 nm. Our approach includes scattering effects beyond Born’s approximation and is remarkably efficient—opening up new routes in ultrafast nanophysics and free-electron laser science.

## Abstract (web summary)

The occurrence of thermodynamically metastable nanoparticles determines the particle growth in nature, but capturing them is experimentally challenging. Barke *et al*. identify the three-dimensional shape of metastable silver nanoparticles in gas phase, characterized by X-ray free-electron laser.

## Graphical abstract

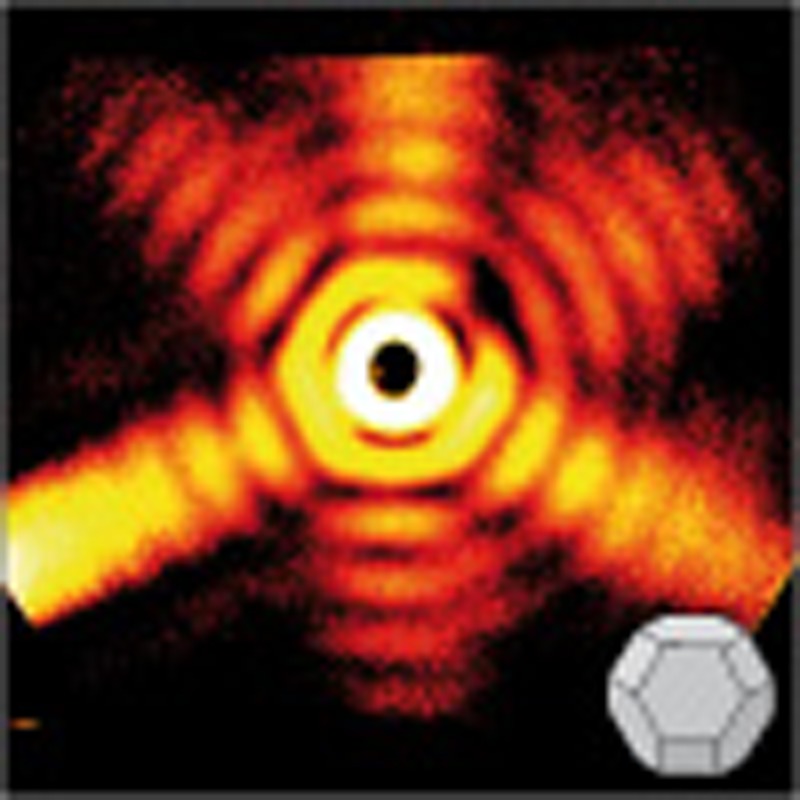

Naturally grown particles exhibit a vast variety of architectures ranging from simple, almost spherical shapes (for example, fog droplets), to highly symmetric polyhedral (for example, clusters or certain viruses) and fascinating, complex geometries such as snowflakes or pollen grains. Studying growth processes of nanostructures addresses the key fundamental question of how geometric structure and stability are determined by the occurrence of thermodynamically metastable shapes during particle formation. An illustrative case is the condensation of metal nanoparticles. Albeit optimal equilibrated shapes can be predicted from energetic considerations such as the Wulff construction^1^, manifold morphologies are frequently reported^2^,^3^,^4^ that are far away from the ideal equilibrium geometries and often beyond theoretical predictability. Conventional microscopy methods have enabled high-resolution imaging of individual particles grown or deposited on surfaces and revealed insight into their geometric properties^3^,^5^. Single free, unsupported particles, however, elude experimental access via microscopy since they cannot be immobilized without an interacting substrate. The current knowledge on the morphology of free metal particles is therefore based on orientation- and ensemble-averaged approaches such as electron diffraction^6^,^7^,^8^, drift measurements^9^ and photoelectron spectrocopy^10^.

An unambiguous experimental morphology characterization of free particles has to face a number of challenges. First, the co-existence of diverse shapes requires circumvention of ensemble averaging by the study of individual particles. Second, to reliably resolve the particle shape irrespective of its orientation, three-dimensional (3D) imaging methods are required. Third, free particle growth is a statistical process such that a specific combination of shape, orientation and size cannot be repeatedly prepared, excluding tomographic techniques^11^,^12^ that rely on multiple measurements of the same object or of equivalent replicas. Considerable efforts have been devoted to obtaining 3D structure information of nanosystems via diffractive imaging with intense femtosecond pulses from X-ray free-electron lasers (X-FELs)^13^,^14^,^15^,^16^,^17^,^18^,^19^,^20^,^21^. In-flight characterization of single nanoparticles by X-ray small-angle scattering has been successfully demonstrated, revealing the effective two-dimensional (2D) projection of the electron density^14^,^16^,^20^. The 3D reconstruction based on single-shot small-angle X-ray scattering data can only be achieved by exploiting additional symmetry information, as was shown for the case of deposited particles^18^. The reconstruction of wide-angle X-FEL scattering has been advocated as an enabling technology for the complete single-shot 3D structure determination of individual nanosystems^15^. However, the short X-FEL wavelengths utilized in previous single-shot in-flight particle imaging studies precluded the detection of sufficient wide-angle signal to apply this technology.

Here we show that this limitation can be overcome by employing soft X-FEL pulses. Based on the resulting single-shot wide-angle scattering images, we demonstrate the identification of symmetry, morphology and orientation of individual gas-phase Ag particles by means of a simple and efficient procedure based on fast simulations. Our approach is complementary to the full reconstruction via phase retrieval methods^14^,^15^,^16^,^18^,^19^,^21^,^22^,^23^ as it allows extracting the relevant 3D structure information from a single scattering pattern of an individual particle without computational inversion of the scattering process, provided that the particles can adequately be described by a parametric geometry model. The reported results provide evidence for metastable shapes of unsupported Ag particles in a so far inaccessible size regime.

## Results

### Wide-angle scattering experiment

**Figure 1:**
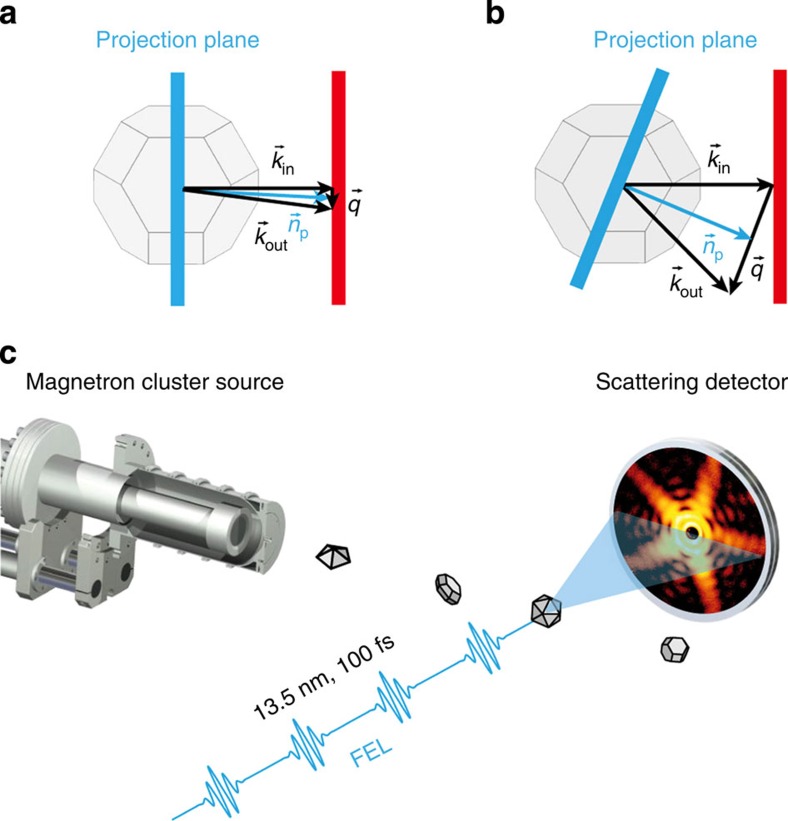
Schematics of the wide-angle scattering experiment. In Born’s approximation, the far-field scattering intensity reads *I*(**q**)∝|∫*ρ*(**r**)*e*^*i***qr**^*d*^3^r|^2^, where *ρ*(**r**) is the scattering density of the particle centered at **r**=0. After decomposing **r** into components parallel (**r**_||_) and perpendicular (**r**_⊥_) to the projection vector **n**_p_=**k**_in_+**q**/2, which is by definition perpendicular to **q**, the intensity can be recast as 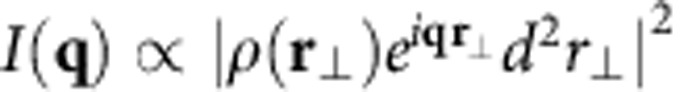, representing the Fourier transform of the projected density *ρ*(**r**_⊥_)=∫*ρ*(**r**)*dr*_||_ on a plane with normal vector **n**_p_. (**a**) For small scattering angles, the approximation **n**_p_||**k**_in_ is valid, which inhibits access to any structural information along this direction. (**b**) The variation of **n**_p_ with **q** for large scattering angles provides access to the 3D properties of the particle. (**c**) In the experiment, single-shot diffraction patterns of silver particles intersecting the FEL photon beam are captured by the 2D detector.

The key experimental requirement for the 3D characterization is the ability to resolve the scattering signal up to large angles. For an illustrative motivation of this requirement, it is convenient to assume validity of the first Born approximation. In the limit of small-angle scattering, the scattered far-field can essentially be described by a 2D Fourier transform of the object’s shape *projected onto a plane* (characterized by normal vector **n**_p_||**k**_in_) perpendicular to the incident beam direction, see Fig. 1a. This follows from the fact that the transfer momentum **q** is small in magnitude (|**q**|≪|***k***_in_|) and therefore essentially perpendicular to the incident wave vector **k**_in_. The resulting intensity distribution (i) reflects only effective 2D information on the object’s density distribution and (ii) is point-symmetric with respect to *q*=0, which impedes unique identification of the target orientation.

These limitations can be overcome by recording scattering under large angles (|**q**|≈|**k**_in_|) because the scattering pattern then reflects 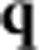 dependent projections of the density (on planes with normal vectors 
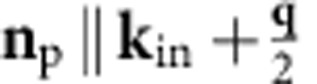
), see Fig. 1b. In a sense, wide-angle scattering enables single-shot tomography as the direction of the projection plane varies with scattering angle *within a single image*.

However, due to the drastic decrease of the scattering intensity *I*(**q**) with increasing |**q**| (Porod’s law), significant signal from the particle shape can in practice be detected only up to a critical angle. Considering near spherical shapes, this angle is roughly proportional to the wavelength and limited to a few degrees when using hard X-ray radiation in the keV range (see Methods). Here we remedy this limitation by employing 90 eV soft X-ray laser pulses to access the wide-angle regime required for the 3D characterization of individual particles.

In the experiments (see scheme in Fig. 1c), silver particles were prepared in a cluster machine^24^ equipped with a magnetron sputtering source and directed into the focus of the 100 fs soft X-ray pulses provided by the free-electron laser (FEL) facility FLASH (see Methods for details). Note that for the employed wavelength of 13.5 nm, the detailed atomic structure is averaged out in the scattering images, simplifying the shape analysis tremendously. Scattering images were captured by a 2D detector with 78° acceptance angle^17^,^25^. A total of 25,000 scattering images with significant intensity have been recorded.

### Scattering patterns and particle morphologies

**Figure 2:**
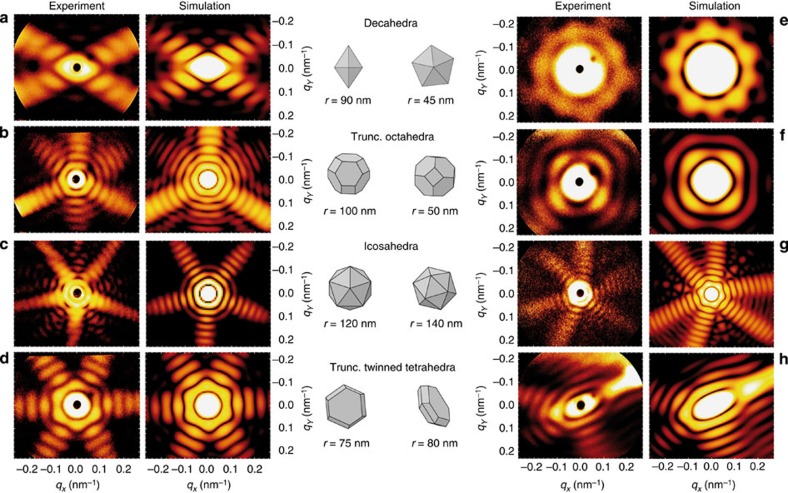
Comparison of measured and theoretical scattering images. (**a**–**d**) Selected experimental scattering patterns of single Ag particles and MSFT simulation results for matched geometries (as indicated). False-colour images show the scattering intensity (logarithmic scale) as function of the transverse components of the scattering vector. The dark spot in the centre of the experimental data originates from a hole in the detector for direct beam transmission. Cluster shapes are drawn as seen from the direction of the incident beam. The size is given by the radius *r* of the polyhedra’s circumscribed sphere. (**e**–**h**) Same cluster shapes as in **a**–**d** imaged at different orientations with respect to the incident beam. Trunc, truncated.

The measured single-particle scattering images (Fig. 2, left column) show highly symmetric patterns with twofold (a), threefold (b), fivefold (c), and sixfold (d) symmetry. The patterns consist of one or more closed ring-like features near the centre followed by discontinuous higher order rings that form streak-like features accompanied by a faint fine structure. Scattering patterns with odd number of mirror axes (Fig. 2b,c), that is, with broken point symmetry, immediately demonstrate that the wide-angle scattering data contains true 3D structure information. Because of substantial absorption of the soft X-ray radiation inside the particles (penetration depth ≈12.5 nm for bulk silver), the morphology identification method must account for scattering effects beyond the Born approximation, which excludes application of conventional iterative reconstruction techniques. We use a simple and efficient multislice Fourier transform (MSFT) algorithm that includes an effective treatment of absorption to calculate scattering images from 3D trial shapes based on a large systematic set of polyhedra (see Methods).

Excellent qualitative agreement between measured scattering images and MSFT results (Fig. 2) is achieved by adjusting size and orientation of the trial model shapes (see Methods for the detailed procedure). In most cases, even fine details are well reproduced, such as the spots in between x-shaped main features in Fig. 2a. Among the morphologies that match the experimental data are decahedra (a), truncated octahedra (b), icosahedra (c) and surprisingly flat hexagonal particles, which correspond to truncated twinned tetrahedra (d). For each of the above shapes, particles with different size and orientation are identified in the data set (see examples in Fig. 2e–h), confirming the repeated occurrence of the identified geometries. Although some of the compact shapes (such as those in Fig. 2b,c) deviate only weakly from a sphere, the scattering images taken for different orientations are extremely diverse (compare Fig. 2b,c to Fig. 2f,g respectively; see Supplementary Movie 1 for a simulation of all high-symmetry orientations of a truncated octahedron). The strong directional and shape sensitivity (even for nearly spherical shapes) demonstrates the 3D capabilities required for the unique identification of particle morphologies. Striking evidence for the transition from small- to wide-angle scattering regimes can be found within a single image for large clusters if it shows broken point symmetry (see Fig. 2b,g). In these cases, the symmetry changes from even, close to the image centre, to odd in the outer regions.

### Benchmark of the MSFT method

**Figure 3:**
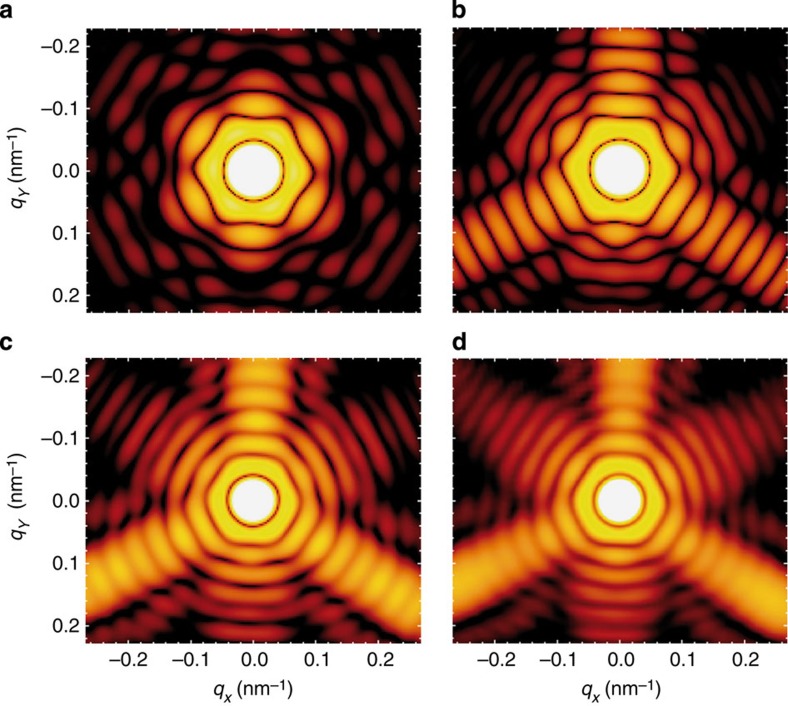
Comparison of different approximation levels. False-colour images show the simulated scattering intensity (logarithmic scale) of a truncated octahedron (cf. Fig. 2b) as function of the transverse components of the scattering vector within different approximations. (**a**) Small-angle approximation corresponding to an effective scattering density projected onto a plane. (**b**) Born’s approximation taking into account the full 3D geometry but no absorption and refraction. (**c**) Same as **b** but including a simplified absorption model. (**d**) Full FDTD simulations using the optical properties of bulk-silver.

To corroborate the reliability of the MSFT method and to elucidate the role of absorption, we have compared theory results for different levels of approximation for the case of the truncated octahedron (cf. Fig. 2b). The resulting data within the small-angle approximation (Fig. 3a), implemented via a 2D Fourier transform of the projected scattering density, predicts point symmetric scattering images (the power spectrum of a real-valued function is symmetric) and fails to resemble the experiment. This point symmetry is lifted in the 3D simulation (Fig. 3b) obtained within Born’s approximation. Comparison to MSFT including effective absorption (Fig. 3c) shows that absorption induces a broadening of scattering features, a relative intensity increase in higher diffraction orders and a reduction of the scattering angle of the first-order intensity maximum. Differences between the MSFT result and that of the full treatment of multiple scattering within the finite-difference time-domain (FDTD) framework (Fig. 3c versus Fig. 3d) are more subtle and in most cases irrelevant for shape identification, justifying the effective absorption treatment in MSFT. Compared with the 3D simulations in Fig. 3b–d, the 2D scattering image in Fig. 3a shows similar features close to the centre but deviates significantly at large scattering angles, illustrating the transition from small-angle to wide-angle scattering within a single image.

### Shape refinement

**Figure 4:**
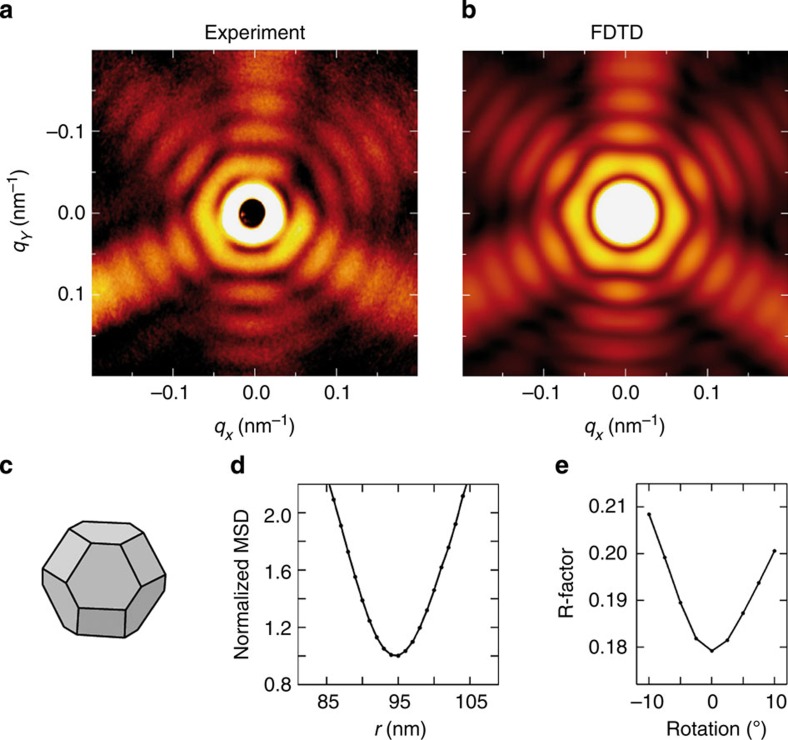
Optimization of model parameters. (**a**) Experimental and (**b**) simulated scattering patterns for a single Ag particle with the optimized shape of a truncated octahedron as depicted in **c** using the FDTD method (false-colour on logarithmic scale). Parameter optimization for truncation and radius was performed by minimization of the mean-squared deviation (MSD) of experimental data from theory as exemplarily shown in **d** for the particle size. The optimal radius is *r*=95 nm with an uncertainty of ±8%, estimated from the curvature of the normalized MSD around the minimum^40^. (**e**) R-factor as a function of rotation angle for the truncated octahedron depicted in **c**. For these calculations, the model shape is rotated away from the optimal orientation in **c** around an axis parallel to the upper edge of the hexagonal front facet.

The MSFT method enables efficient shape identification from a finite set of trial shapes and yields a rough estimate of the parameters of the respective geometry model. A more accurate description of the scattering process via FDTD simulations offers further refinement of the free parameters by direct minimization of the mean-squared deviation of calculated and experimental scattering patterns (see Methods for technical details). This is exemplarily illustrated in Fig. 4 for the truncated octahedron from Fig. 2b, yielding even better agreement between experiment and simulation (R-factor ≈0.18, see Fig. 4e). The resulting shape (Fig. 4c) was obtained after optimizing the degree of truncation and the radius of the particle, leading to a slightly reduced value of the predicted radius of *r*=95 nm (Fig. 4d) and a relative truncation of 0.39, as compared with the MSFT estimate of *r*=100 nm obtained under the assumption of an ideal Archimedean shape with a truncation of 1/3. This scheme can be extended to other parameters such as orientation (see Fig. 4e) or shape asymmetries to obtain precise information on the geometry of the individual nanoparticle.

## Discussion

The variety of particle structures derived from the scattering images in Fig. 2 demonstrates that the motives are much richer than expected from thermodynamic considerations in the investigated size regime. Similar morphologies as those found in this work were reported for preparations involving chemical reactions^3^, physically evaporated^2^,^26^ or cluster-beam deposited^27^ particles, providing evidence for the general existence of such shapes. The particles in these studies were, however, *supported* by a substrate and are not imaged in the free beam. In the absence of a surface, only truncated octahedra can be derived by free surface energy minimization of particles with face-centred cubic (fcc) lattice structure. Decahedral and icosahedral geometries represent metastable states whose facets cannot be constructed from low-index surfaces of a single fcc crystallite. Particularly striking is the case of unsupported icosahedral particles, which are stable for small clusters but have been predicted to undergo a transition to fcc-derived structures already for sizes as low as a few hundred atoms^28^. Previous observations of icosahedral clusters considerably larger than that (a few 10^6^ atoms) have been explained by a non-equilibrium growth process, where isomers being favoured for small sizes provide seed shapes that persist in subsequent stages of particle formation^2^,^7^, often resulting in multiple twinned species^3^. The current results demonstrate the existence of free metastable nanoparticles up to much larger radii of ≈150 nm (≈10^9^ atoms). The identified highly symmetric metastable shapes reveal that free Ag nanoparticles retain a structural memory of early formation stages up to this so far unexplored size range. This conclusion is corroborated by the observation of strongly oblate particles with aspect ratios of about four (Fig. 2h). The 30–40% larger surface of such shapes substantially increases the surface energy when compared with minimal energy structures (for example, truncated octahedral). In contrast to supported particles, where anisotropic shapes can result from cluster–surface interactions^29^,^30^, the strongly anisotropic free metal particles observed here provide evidence for pronounced symmetry breaking solely induced by the seed structures, in surprising analogy to the formation of ionic or water crystals like salt or snowflakes.

Retrieving the 3D morphology of free nanoparticles from single wide-angle scattering patterns opens up new routes for interdisciplinary research. The efficient and simultaneous determination of size, shape and orientation enables systematic high-throughput studies of particle properties. Applied to time-resolved experiments, the shape-specific evolution of growth, structural phase transitions and relaxation phenomena become accessible, with implications for various fields including molecular and atmospheric physics, material science, chemistry and astrophysics. Combined with femtosecond pump-probe schemes, single-shot imaging promises new insight into the ultrafast dynamics of free nanoparticles, such as collective electron^31^ and nuclear^32^ motion or non-equilibrium melting processes. Moreover, the sensitivity to the complex index of refraction may provide access to ultrafast changes of optical and electronic properties of nanoparticles^17^.

## Methods

### Particle source

Silver particles were produced by a cluster beam machine^24^ equipped with a magnetron sputtering source, operated with Ar and Xe at a pressure of the order of ≈1 mbar. The aggregation section was cooled with liquid nitrogen. No mass selection or ion optics have been used. The clusters were guided into the differentially pumped main chamber through a conical skimmer with 3 mm inner diameter.

### Scattering experiment

About 70 cm behind the source, the beam density was low enough to ensure a single nanoparticle at a time in the focal volume (focus size≈20 μm) of the FEL FLASH at DESY in Hamburg. The power density of the FEL pulses has been estimated from the highest observed charge state of atomic Xe. Typically, Xe^11+^ is found which corresponds to 10^14^-10^15^ W cm^−^^2^ at a pulse length of ≈100 fs (refs 33, 34). The scattering patterns were recorded shot-to-shot (10 Hz repetition rate) with an imaging detector as described in refs 17, 25, covering scattering angles from *θ*≈3° to 39° over a 2π azimuth. To avoid detector damage from the direct FEL beam, all detector components have a centre hole. The tilt angle of the detector microchannels results in a depletion artefact visible at (*q*_*x*_, *q*_*y*_)≈(0.6, –0.4) nm^−1^ in the scattering images (for example, Fig. 2d). Due to the weak focus conditions (Rayleigh length: some mm; wavelength: 13.5 nm), the description as a plane wave is justified for the incident beam. In order to maintain single-particle conditions in the focal volume^35^, the hit rate was kept below 10%. Most of the 25,000 scattering patterns with significant signal (from a total of 300,000 recorded images) originated from small clusters resulting in no fringes or from agglomerates of two or more particles (for typical signatures see ref. 35). About 1,000 images were suited for further analysis, for about 100 of them, the particles’ shape, size and orientation have been uniquely identified in this work. The most common symmetric morphologies are truncated octahedra, flat hexagonal shapes, decahedra and isosahedra. Due to manual selection of scattering images for identification, the obtained variety of shapes does not necessarily reflect the actual statistics present in the particle beam.

### Experimentally accessible angular range

Within Born’s approximation and under the assumption of free electrons (scattering factors of unity), the scattering cross-section 
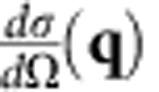
 for a homogeneous sphere, and therefore also the scattering intensity *I*(*q*) measured at a fixed distance, is independent of the photon wavelength up to trivial polarization effects. As the envelope of the resulting fringe pattern rapidly decreases with the transfer momentum *q* (Porods law: *q*^−4^), the experimentally accessible *q*-range is restricted to a maximum value *q*_crit_ (limited by dynamic range of the detector, background noise, incoherent scattering and so on). The relation between *q* and scattering angle *θ* is 
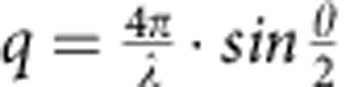
, hence the angular range onto which the accessible part of the scattering pattern is mapped scales (in first-order approximation) as *θ*_crit_∝*λ*·*q*_crit_. Therefore, the wide-angle scattering regime is easily accessible for soft X-ray radiation used in our experiment (*λ*=13.5 nm), in contrast to hard X-rays (*λ*<1 nm), where the detectable signal is restricted to an angular range of a few degrees for realistic experimental conditions.

### Observed scattering features

Independent of shape, the scattering images show a number of quasi-continuous, often interrupted rings near the centre (for example, Fig. 2b,c), whose distance Δ|**q**_||_| directly reflects the particle size (for example, for a sphere via Δ|**q**_||_|=*π*/*r* due to Mie scattering^17^). This enables a useful size estimate, almost irrespective of other parameters. Note that while the maxima of the radial intensity oscillations for a sphere are strictly equidistant as function of *q*, their period length decreases towards the edges of the scattering images when plotting against *k*_*x*_=*q*_*x*_ and *k*_*x*_=*q*_*x*_. For non-spherical shapes, the ‘ring distance’ Δ|**q**_||_| at large |**q**_||_| depends on the direction in the (*q*_*x*_, *q*_*y*_) plane, as is most apparent for strongly oblate clusters such as the decahedron (Fig. 2a) or the truncated twinned tetrahedron (Fig. 2h). The breakup of rings into radial oscillating streaks indicates the transition from signatures of the coarse shape (for example, compact versus flat) towards sensitivity to the arrangement of facets, edges and vertices at higher diffraction orders. Bright streaks in the scattering image can be attributed to the diffraction of facets that are aligned almost parallel to the incident photon beam. The direction and intensity of the observed streaks thus enables an efficient preselection of relevant morphological candidates for the matching process to simulation data.

### Multislice Fourier transfom method

The model electron density distributions used for calculating the scattering patterns are divided into stacks of 2D slices whose normal vectors are oriented parallel to the incident photon beam. This is similar to techniques previously applied to electron scattering^7^,^36^,^37^, as well as to soft X-ray diffraction of supported particles^38^. The final scattering intensity corresponds to the modulus square of the phase-correct sum of the 2D Fourier transforms of the slices. Polarization properties have been included via an angle-dependent differential Thomson cross-section. Material properties are approximately taken into account by reducing the scattering amplitude as a function of propagation through the material according to Beer–Lambert’s law. Compared with the direct field summation from an arrangement of point scatterers, the MSFT method accelerates the computation by two orders of magnitude for typical particles investigated here.

### Shape identification

The procedure for finding the size, shape and orientation of a particle from a single scattering image is as follows. The observed features are first qualitatively compared with simulation results based on a finite set of model shapes that consists of all Platonic and Archimedean shapes up to 32 facets, derived geometries obtained via truncation and some additional shapes described in the literature as candidates for deposited metal particles (see for example, refs 2, 3, 26, 27). We used homogeneous density distributions for the simulations, but inhomogeneities arising from impurities or core shell structures are accessible if they induce distinct features in the wide-angle scattering image. The priority for judging the quality of this comparison is given to the number, direction, and shape of the main streaks. For each shape, all inequivalent orientations are tested along high-symmetry directions or in coarse steps of typically 5°–10° around two axes (the last axis corresponds to a trivial 2D rotation of the scattering image as a whole). If qualitative agreement is achieved, an approximate particle size is determined from the distance Δ|**q**_||_| of the higher-order diffraction rings. The model shape is then rotated until best agreement between simulation and experiment is achieved. In a last step, the precise particle size is adjusted by matching the period of simulated features in radial **q**_||_ direction to the experimental data^19^. Since the duration for calculating one MSFT pattern is about a second on a standard desktop computer, this procedure is surprisingly efficient, despite the need for a large quantity of simulations per experimental scattering image. Among the set of model shapes described above each identified shape is unique in that any other model results in significant qualitative discrepancies between experimental and simulation results. Only in the case of extremely flat platelets (see Fig. 2d,h), the exact orientation of the small-side facets cannot be specified since they do not give rise to pronounced features in the diffraction pattern. The important observation of the large aspect ratios, however, remains unaffected.

### Finite difference time domain simulations

For accurate refinement of model parameters describing the shape and for benchmarking the MSFT approach, we performed FDTD simulations of the wide-angle scattering. Therefore, the model shapes are sampled on a cubic, equidistant grid with spatial resolution Δ*x*=1.0 nm and 959 × 959 × 319 cells, where the shortest dimension corresponds to the propagation direction of the soft X-ray radiation. The complex permittivity of bulk silver for the considered wavelength of 13.5 nm was used^39^. The corresponding attenuation length is *λ*_abs_≈12.5 nm for bulk silver. Maxwell’s equations are solved for a plane incident wave on a staggered grid (Yee scheme) with absorbing boundary conditions (uniaxial perfectly matched layer) using the FDTD implementation of ref 31. The complex scattered electric near-fields are extracted on a plane with normal vector parallel to the optical axis placed closely behind the target structure. The scattered far field intensity distributions are obtained using a near-to-far-field transformation of the continuous wave solution. A typical result of the FDTD method is compared with MSFT in Fig. 3c,d.

### Refinement of parameters describing the model shape

Refinement of the model shape is done by optimizing additional parameters such as orientation, truncation and size. An upper bound for the error of the parameters specifying the model shape can be estimated via the mean-square deviation of experimental and simulated scattering images. Such a quantitative comparison requires FDTD calculations to accurately include the material properties and knowledge of the detector response as a function of intensity, which is in general nonlinear and may be described by a power behaviour (in our case, the exponent is close to 1/3). The example in Fig. 4 results in a near-parabolic evolution of the deviation as a function of radius, from which the parameter uncertainty (that is, the standard deviation of a parameter in a non-linear fit, see ref 40) *σ*_*r*_=7 nm can be estimated. This calculation has been performed after optimization of the truncation, hence the radius slightly differs (≈5 nm) from the one given in Fig. 2b, where the latter reflects the best MSFT estimate. We note that the reduced radius has only minor effect on the particle volume because of the specific definition of the radius as that of a circumscribed sphere. The R-factor in Fig. 4e was calculated via *R*=∫∫|*I*_exp_−*I*_calc_|*dq*_*x*_*dq*_*y*_/∫∫*I*_exp_*dq*_*x*_*dq*_*y*_, where *I*_exp_ and *I*_calc_ are the experimentally observed and FDTD-calculated intensities in the scattering images, respectively.

## Author contributions

H.H., S.B. and I.B. designed the setup of the cluster source, D.R., L.F., M.S., M.A. and C.B. designed and prepared the scattering setup, D.R., L.F., M.S., I.B., H.H, C.B. and S.S. performed the measurements, R.T. took care of the FLASH laser, C.P. and T.F performed the FDTD calculations, K.-H.M.-B., T.M., D.R., T.F., C.P., H.H. and I.B. analyzed and discussed the results, I.B. performed the MSFT calculations and wrote the manuscript with the input of all co-authors.

## Additional information

**How to cite this article:** Barke, I. *et al*. The 3D-architecture of individual free silver nanoparticles captured by X-ray scattering. *Nat. Commun.* 6:6187 doi: 10.1038/ncomms7187 (2015).

## Supplementary Material

Supplementary Movie 1 The video shows simulated scattering images from a truncated octahedron with radius 120 nm for varying particle orientations as predicted by the multislice calculation. Absorption and refraction are neglected. The actual particle orientation as seen by the incident soft X-ray beam is displayed at the lower left corner.
